# 12-O-Tetradecanoylphorbol-13-Acetate Induces Up-Regulated Transcription of Variant 1 but Not Variant 2 of *VIL2* in Esophageal Squamous Cell Carcinoma Cells via ERK1/2/AP-1/Sp1 Signaling

**DOI:** 10.1371/journal.pone.0124680

**Published:** 2015-04-27

**Authors:** Xiao-Dan Zhang, Jian-Jun Xie, Lian-Di Liao, Lin Long, Yang-Min Xie, En-Min Li, Li-Yan Xu

**Affiliations:** 1 The Key Laboratory of Molecular Biology for High Cancer Incidence Coastal Chaoshan Area, Medical College of Shantou University, Shantou 514041, Guangdong, P.R. China; 2 Institute of Oncologic Pathology, Medical College of Shantou University, Shantou 514041, Guangdong, P.R. China; 3 Department of Biochemistry and Molecular Biology, Medical College of Shantou University, Shantou 514041, Guangdong, P.R. China; 4 Department of Experimental Animal Center, Medical College of Shantou University, Shantou 515041, P. R. China; University of Hong Kong, HONG KONG

## Abstract

The membrane-cytoskeleton link organizer ezrin may be the most “dramatic” tumor marker, being strongly over-expressed in nearly one-third of human malignancies. However, the molecular mechanisms of aberrant ezrin expression still need to be clarified. Ezrin, encoded by the *VIL2 *gene, has two transcript variants that differ in the transcriptional start site (TSS): V1 and V2. Both V1 and V2 encode the same protein. Here, we found that 12-O-tetradecanoylphorbol-13-acetate (TPA) induced over-expression of human *VIL2 *in esophageal squamous cell carcinoma (ESCC) cells. Furthermore, *VIL2 *V1 but not V2 was up-regulated after TPA stimulation in a time-dependent manner. AP-1 and Sp1 binding sites within the promoter region of *VIL2 *V1 acted not only as basal transcriptional elements but also as a composite TPA-responsive element (TRE) for the transcription of *VIL2 *V1. TPA stimulation enhanced c-Jun and Sp1 binding to the TRE via activation of the ERK1/2 pathway and increased protein levels of c-Jun, c-Fos, and Sp1, resulting in over-expression of *VIL2 *V1, whereas the MEK1/2 inhibitor U0126 blocked these events. Finally, we showed that TPA promoted the migration of ESCC cells whereas MEK1/2 inhibitor or ezrin silencing could partially inverse this alteration. Taken together, these results suggest that TPA is able to induce *VIL2 *V1 over-expression in ESCC cells by activating MEK/ERK1/2 signaling and increasing binding of Sp1 and c-Jun to the TRE of the *VIL2 *V1 promoter, and that *VIL2 *is an important TPA-induced effector.

## Introduction

Ezrin is a member of the Ezrin-Radixin-Moesin (ERM) cytoskeleton-associated protein family, which primarily acts as physical and functional links between the plasma membrane and cytoskeleton [1,2]. Ezrin is essential for many cellular processes including the determination of cell polarity, formation of surface structure, cell adhesion, cell-cell interaction, cytokinesis, migration/invasion, and signal transduction [3,4]. The aberrant expression of ezrin is associated with the development and progression of several human cancers, such as hepatocellular carcinoma [5,6], lung cancer [7,8], breast carcinoma [9], pancreatic adenocarcinoma [10], colon cancer [11], osteosarcoma [12,13], rhabdomyosarcoma [14,15], and endometrial cancer [16]. We previously showed that the overexpression of ezrin in esophageal squamous cell carcinoma (ESCC) may be involved in the growth and invasiveness of ESCC cells and ezrin expression can serve as a biomarker that predicts the prognosis of ESCC patients [17,18]. However, although much is known about the functional role of ezrin in cancer development and progression, the biochemical mechanism of ezrin up-regulation has not been thoroughly investigated.

Being encoded by the *VIL2* gene, ezrin has two transcript variants—variant 1(V1) and variant 2 (V2)—that differs in the transcriptional start site, but both V1 and V2 encode the same protein, termed as ezrin. Previous studies showed that a range of cytokines, including interleukin 2 (IL-2), IL-8, IL-10, and insulin-like growth factor 1, inhibited ezrin expression in human colon cancer cells, whereas epidermal growth factor and IL-11 enhanced ezrin expression [19]. TNFα enhanced both ezrin expression and phosphorylation in human endothelial cells, which promoted its nuclear translocation [20]. In mouse rhabdomyosarcomas, *VIL2* has been suggested to be a downstream target of the homeoprotein transcription factor sineoculis homeobox homolog 1 (Six1), which binds to the *VIL2* promoter and regulates its transcription [15,21]. Also, *VIL2* is regulated by epigenetic modifications such as histone modification and DNA methylation in its promoter region, and up-regulation of *VIL2* associated with histone ‘active codes’ (i.e., acetyl-H3-K9 and tri-methyl-H3-K4) and unmethylated CpG islands within its promoter region [14]. In ESCC cells, we previously found specificity protein 1 (Sp1) and activator protein 1 (AP-1, a c-Jun/c-Fos heterodimer) co-regulated *VIL2* promoter activity and ezrin basic expression [22]. Furthermore, 12-O-tetradecanoylphorbol-13-acetate (TPA), a tumor promoter, could lead to the malignant transformation of human embryonic esophageal mucosa cells to ESCC cells, in which ezrin was overexpressed obviously, suggesting TPA might be an inducer of *VIL2* overexpression in ESCC cells [23,24]. These findings of the observation of dramatic overexpression of ezrin in various cancer cells prompt us to explore the induced mechanisms of ezrin over-expression in ESCC.

Herein, we firstly investigated the effects of HGF, IL-6, PDGF, testosterone, TGF, TPA and VEGFC stimulation on *VIL2* transcription in ESCC cells, and found that TPA could up-regulate the transcription of *VIL2* V1, but not V2, through ERK1/2/AP-1/Sp1 signaling, resulting in the enhancement of cell mobility.

## Materials and Methods

### Reagents and antibodies

Plasmids pGL3-basic and pRL-TK, as well as the MEK1/2-specific inhibitor U0126, were purchased from Promega. Antibodies against Sp1 (rabbit monoclonal antibody, 1:1000 dilutions), c-Jun (rabbit polyclonal antibody, 1:500 dilutions), c-Fos (rabbit polyclonal antibody, 1:500 dilutions), ERK1/2 (rabbit polyclonal antibody, 1:1000 dilutions), and β-actin (mouse monoclonal antibody, 1:1000 dilutions) were purchased from Santa Cruz Biotechnology. Antibodies against p-ERK1/2 (Thr202/Tyr204) (rabbit monoclonal antibody, 1:1000 dilutions), T567 ezrin (rabbit monoclonal antibody, 1:1000 dilutions) were purchased from Cell Signaling (Beverly, MA) and the ezrin antibody was purchased from Neomarker (mouse monoclonal antibody, 1:500 dilutions). TPA, dimethyl sulfoxide (DMSO), and β-tubulin antibody (mouse monoclonal antibody, 1:1000 dilutions) were purchased from Sigma. All other reagents were of analytical reagent grade.

### Constructs

**Fig 1:**
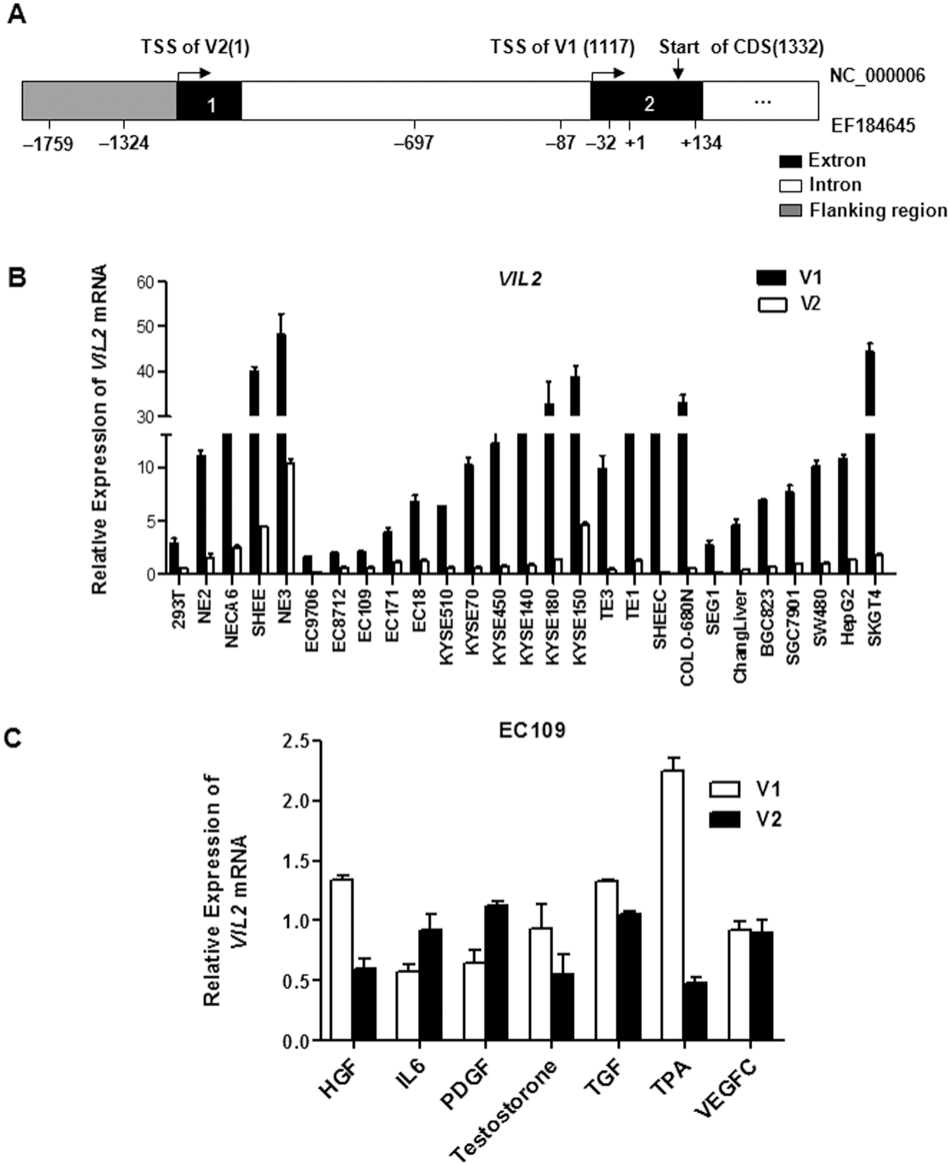
Expression of the *VIL2* transcription variants in cell lines. (A) The genomic structure of *VIL2* gene. TSS, transcriptional start site; CDS, coding sequencing. The gene information was adapted from NCBI database (Accession number: NC_000006) and Genbank database (Accession number: EF184645). (B) qRT-PCR was used to address the expression of the *VIL2* transcription variants in a panel of cells lines. Each bar represents the mean ± SD. (C) Alteration of *VIL2* V1 or V2 mRNA level in response to inducers treatment. The total RNA extract (1 μg) from ESCC cells treated with or without inducers for 24 h, and subjected to qRT-PCR analysis using gene-specific primers. The relative value from the control group was considered equal to one arbitrary unit. Each point represents the mean ± SD. The data are representative of at least two independent experiments.

The parent reporter vector for all *VIL2* 5′-flanking region constructs was pGLB-hE(-1759/+134), which was cloned in our previous work [22]. The entire length of the sequence was from +134 to -1759 base pairs (bp) of the transcribed human *VIL2* sequence (GeneBank No. EF184645). pGLB-hE(-1695/-1148), pGLB-hE(-1229/+134), and pGLB-hE(-1119/+134) were generated by polymerase chain reaction (PCR) using the primers shown in Table 1 and subcloned into the pGL3-basic vector. The -1695/-1148 sequence, from +50 to -498 bp of the transcriptional start site of human *VIL2* V2, was considered the potential promoter region of *VIL2* V2 (Fig 1A). pGLB-hE(-1324/+134), pGLB-hE(-87/+134), pGLB-hE(-87/-134)Sm, pGLB-hE(-87/-134)Am, and pGLB-hE(-87/-134)Sam luciferase reporter plasmids were also generated in our previous work [22].

**Table 1:** Primers used in this study.

Primers	Sequences	Names of constructs
**Primers for luciferase-reporter gene constructs**		
EZR-1229F	5′-CGGGGTACCCGCACCTCACAGGTCGGGAGCT-3′	pGLB-hE(-1229/+134)
EZR-1119F	5′-CGGGGTACCCGGGGAGCACACGGAGCACT-3′	pGLB-hE(-1119/+134)
EZR+134R	5′-CCCAAGCTTTCGGTTTCTGGTGAGTATCCTCGATCCC-3′	
EZR-1695F	CCGCTCGAGCCGCAGCAAATTCTACTGGCCC	pGLB-hE(-1695/-1148)
EZR-1148R	5′-CGCGGATCCGCTGCCCGCGCTCCCAAAAG-3′	
**Primers for qRT-PCR**		
EZR1Q-F	5′-GCGGGCGCTCTAAGGGTTCT-3′	
EZR1Q-R	5′-ACTCGGACATTGATTGGTTTCGGC-3′	
EZR2Q-F	5′-CTTTTGGGAGCGCGGGCAGC-3′	
EZR2Q-R	5′-AGACGCTGTCCCAACCCGGC-3′	
F-actin	5′-CAACTGGGACGACATGGAGAAA-3′	
R-actin	5′-GATAGCAACGTACATGGCTGGG-3′	
**Primers for ChIP**		
*VIL2*-95/-76	5′-CTCCCCATGCCCGCAGTGCT-3′	
*VIL2*-123/-99	5′-GGTGAGTATCCTCGATCCCCGAAAA-3′	
Negative-F	5′-ATGGTTGCCACTGGGGATCT-3′	
Negative-R	5′-TGCCAAAGCCTAGGGGAAGA-3′	

F: forward primer; R: reverse primer.

Cutting sites are underlined.

### Cell culture and transfection

Cell lines used in this study and related cell culture general information were listed in the S1 Table. Mainly, EC109 cells were cultured in Dulbecco's modified Eagle’s medium (GIBCO) supplemented with 10% new-born bovine serum (Excell Biology Inc). KYSE180 cells were maintained in 1640 medium (Thermo Scientific) containing 10% fetal bovine serum (Thermo Scientific). Both cell lines were incubated at 37°C in a humidified atmosphere containing 5% CO_2_ and maintained in media supplemented with penicillin-G (100 units/ml) and streptomycin (100 μg/ml). EC109 and KYSE180 cells were seeded into 12-well plates at the density of 2×10^5^ cells per well and cultured for 12–24 h until grown to 50–80% confluence. In the experiments of inducers treatment, cells were starved in serum-free medium overnight followed by incubation with HGF (50ng/ml, Invitrogen), IL6 (10ng/ml, Invitrogen), PDGF (50ng/ml, Invitrogen), testosterone (10 nM, Baiyunshan), TGF (5 ng/ml, Invitrogen), VEGFC (10 ng/ml, Invitrogen) or TPA (40 ng/ml, Sigma) for another 24 h, respectively. In the experiments with inhibitor, U0126 (Promega) was added to the medium 1 h before DMSO or TPA (10ng/ml) treatment. Cells were then harvested and used for further analysis. In the siRNA-mediated knockdown experiments, siRNA targeting ezrin (siEzrin, 5′-CAGGACUGAUU GAAUUACGGAtt-3′) was transfected into ESCC cells. These cells were harvested 48 h later and used for further analysis.

### RT-PCR and qRT-PCR assay

Total RNA was extracted and purified using TRIzol (Invitrogen) according to the manufacturer’s protocol. Subsequently, 1 μg total RNA was reverse transcribed into cDNA using PrimeScript RT reagent Kit with gDNA Eraser (TaKaRa) according to the manufacturer’s protocol. The target genes of *VIL2* V1, *VIL2* V2, and β-actin were amplified with the specific primers shown in Table 1. Quantitative RT-PCR(qRT-PCR) was performed in 10 μl total reaction mixture; denaturation occurred at 95°C for 30 s followed by 40 cycles with 5 s at 95°C and 30 s at 64°C. Relative levels of the *VIL2* V1 or V2 mRNA were normalized to that of β-actin mRNA. The relative value from the DMSO-treated control group was considered equal to one arbitrary unit. All experiments were repeated at least three times with n = 3 samples per experiment.

### Dual-luciferase reporter assay

Cells were seeded into 96-well plates at the density of 1.5×10^5^ cells/ml and cultured for 12–24 h until grown to 50–80% confluence. They were then co-transfected with a *firefly* luciferase-expressing plasmid (1μg), and a *renilla* luciferase-expressing plasmid (20ng) (pRL-TK, Promega) as an internal control, using Attractene Transfection Reagent (QIAGEN) according to the manufacturer’s protocol. Twenty-four hours later, cells were treated with TPA for another 24 h and then harvested using passive lysis buffer (Promega). Luciferase activity was analyzed using the Dual-Luciferase Reporter Assay System (Promega) according to the manufacturer’s protocol. Values for each group are expressed as the mean ± standard deviation (SD) for three separate experiments.

### Electrophoretic mobility shift assay

NE-PER Nuclear and Cytoplasmic Extraction Reagents, LightShift Chemiluminescent Electrophoretic mobility shift assay (EMSA) Kit, and Chemiluminescent Nucleic Acid Detection Module were purchased from Thermo Scientific. Purified biotin-labeled probes were purchased from Sangon Biotech. Cells were treated with DMSO or TPA, and then nuclear extracts were isolated. For binding assays, 3 μg nuclear extracts were incubated with 20 fmol probes at room temperature for 20 min in 20 μl reaction buffers. Electrophoreses, transfer to a nylon membrane, and UV cross-linking were performed according to the manufacturer’s protocol. For confirming the specificity of the transcription factor binding to the probe, the antibodies against c-Jun or Sp1 (Santa Cruz) and 3 μg nuclear extracts were incubated for 20 min at room temperature before adding the purified biotin-labeled probe for another 20 min at room temperature. Oligonucleotide probes used in EMSA were named as wild-type (WT), Am, Sm, or Sam, and their sequences corresponded to the human *VIL2* V1 promoter region from -87 to -46 bp (Table 2).

**Table 2:** Probes for EMSA.

Probe Name	Sequence	Substituted sites
WT	5′-GCCCGCAGTGCTGGGCGGGGCGCTGACTCACCCGGGCCCGGG-3′	wild-type sequence
Sm	5′-GCCCGCAGTGCTAATATTTGCGCTGACTCACCCGGGCCCGGG-3′	Sp1 binding site
Am	5′-GCCCGCAGTGCTGGGCGGGGCGCGTCGGATCCCGGGCCCGGG-3′	AP-1 binding site
SAm	5′-GCCCGCAGTGCTAATATTTGCGCGTCGGATCCCGGGCCCGGG-3′	both Sp1 and AP-1 binding sites

Substituted sites are underlined.

### Chromatin Immunoprecipitation (ChIP)

Cells were grown to 60–70% confluence in 10-cm plates. After starved in serum-free medium for 12 h, cells were treated with or without TPA for another 6 h. ChIP-IT kit (Active Motif) was used in Chromatin Immunoprecipitation assay according to the manufacture's protocol. Briefly, cells were cross-linked with 1% formaldehyde for 10 min at room temperature and then washed and harvested in ice-cold PBS containing protease inhibitor PMSF (500 μM). After sonication, sheared chromatin was pre-cleared with protein G beads and then 10 μl of the pre-cleared chromatin was transferred to a microcentrifuge tube, which was set as “Input DNA”. Then sonicated chromatin preparation was incubated overnight with 3 μg of antibodies against IgG, Sp1 and c-Jun, respectively. Purified DNA was used for PCR analysis. The human *VIL2* promoter-specific primers and negative control primers were used in our previous work [22], and all of them were shown in Table 1.

### Western blotting analysis

Total cell lysates collected from ESCC cells were prepared in 1× Laemmli Sample Buffer (Bio-Rad). Equal amounts of protein were loaded and separated by SDS-PAGE and transferred to a PVDF membrane (Roche). Blots were blocked in blocking buffer and incubated with antibodies against Sp1, c-Jun, c-Fos, p-ERK, ERK, ezrin, T567 ezrin or β -actin. Specific immunoreactive bands were detected by luminol reagent.

### Cell migration assays

Wound-healing assay and Boyden chamber cell migration assay were used to evaluate cell migration ability. For wound-healing assays, cells were inoculated onto the plates and grown to confluence for 24 h. And then the cells were starved in serum-free medium for 12 h, and a scratch made across the monolayer using a sterile pipette tip. Wound closure information was imaged at 0, 6, 12, 24 and 48 h with a 40×objective (Leica), respectively. In Boyden chamber cell migration assay, 4 × 10^4^ cells were seeded onto the top chamber and the bottom chamber was filled with medium containing 10% fetal calf serum. The membranes were fixed and stained by Giemsa reagent 24 h later and migrated cells were quantified by counting 10 random fields under a light microscope (40×). The mean value was calculated from data obtained from three separate chambers.

### Statistical analysis

Statistical analysis was performed using SPSS software. All data are reported as mean ±SD. Student’s *t*-tests were used to compare groups. Statistical significance was set at *p*< 0.05.

## Results

### TPA induces the transcription of *VIL2* V1 but not V2.

**Fig 2:**
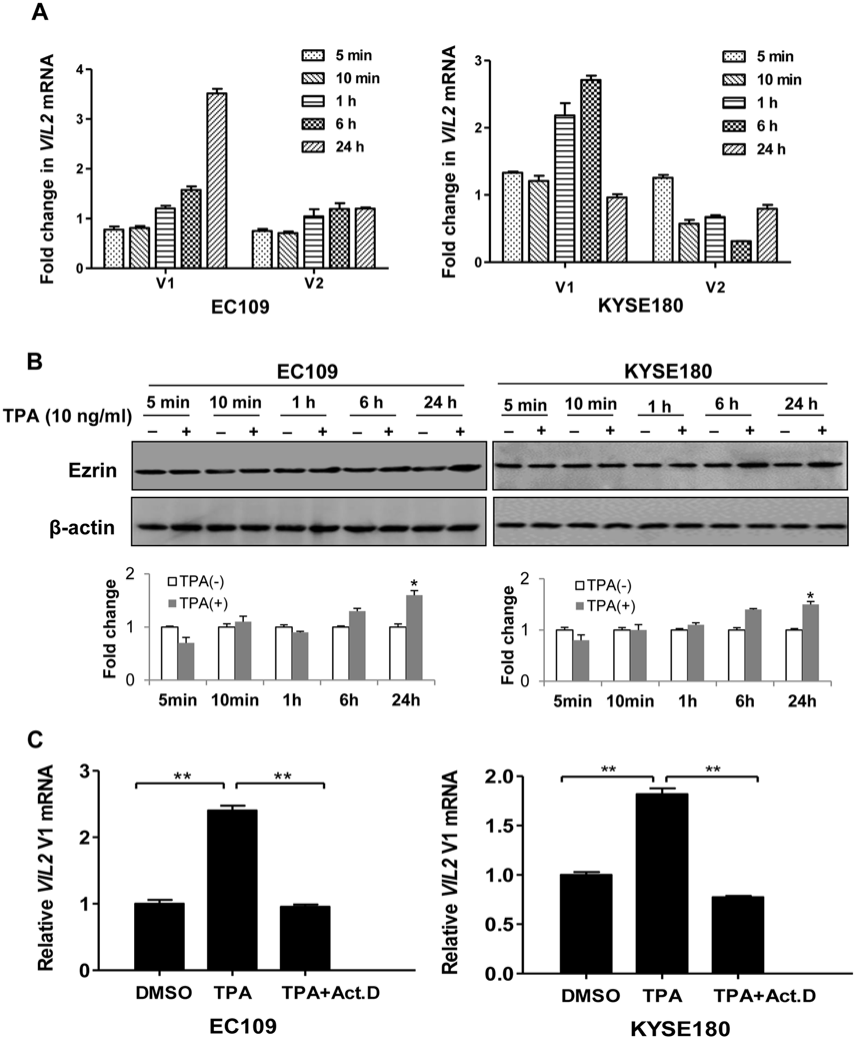
TPA increased transcriptional expression of human *VIL2* transcript variant V1 but not V2. (A) Alteration of *VIL2* V1 or V2 mRNA level in response to TPA treatment. The total RNA extract (1 μg) from ESCC cells treated with DMSO or TPA (10 ng/ml) for various time were reversely transcribed, and subsequently use qRT-PCR assay to detect the change. The relative value from the DMSO-treated control group was considered equal to one arbitrary unit. Each point represents the mean ± SD, n≥3. (B) EC109 and KYSE180 cells were stimulated with DMSO or TPA (10 ng/ml) for the indicated time after starvation for 12 h. Western blot was used to determine the protein level of ezrin upon TPA treatment. Signal intensity for the expression of ezrin was quantified by densitometric scanning and normalized by internal control (β-actin). The data represent the means ± SD of triplicate experiments. (C) Relative RT-PCR analysis. The total RNA extract was prepared from EC109 and KYSE180 cells after stimulation with DMSO or TPA (10 ng/ml) for 6 h, or pretreated with actinomycin D (1 μg/ml) for 1 h before TPA was added (10 ng/ml). The target genes were examined using the specific primers. The densitometry values for *VIL2* V1 levels were normalized to the values for β-actin and then presented relative to that of the DMSO-treated control, which was set as 1. The results of a representative experiment were presented as mean ± SD of the two independent samples. *p< 0.05 or **p< 0.01.

Because human *VIL2* has two variants (V1 and V2, Fig 1A), we first examined their transcription levels in a panel of 27 human cell lines using qRT-PCR. Both V1 and V2 were expressed in all cell lines detected, the mRNA level of V1 was higher than V2 (Fig 1B). To explore the different induced-expression of *VIL2* variants, HGF, IL-6, PDGF, testosterone, TGF, VEGF and TPA were used. Results of qRT-PCR assay revealed that TPA showed the greatest induction of the transcription of *VIL2* V1 among those inducible factors, whereas it did not affect the expression of *VIL2* V2 (Fig 1C). To further confirm this finding, two cell lines were treated with or without TPA (10 ng/ml) for different time points. Results showed that TPA treatment increased the mRNA level of *VIL2* V1 in a time-dependent manner, but not V2 (Fig 2A). Induced expression of TPA on ezrin protein level was also addressed by using western blotting (Fig 2B). Moreover, to explore whether the increase of *VIL2* V1 mRNA level and ezrin protein expression trigged by TPA treatment was linked to transcriptional regulation, we then treated cultured cells with TPA (10 ng/ml) or the combination of TPA (10 ng/ml) and actinomycin D (10 μg/ml, a transcription inhibitor) for 6 h and determined the level of *VIL2* V1 by qRT-PCR. *VIL2* V1 mRNA was up-regulated after treatment with TPA, but decreased after the combined treatment of actinomycin D and TPA (Fig 2C). These results strongly suggest that TPA-induced elevation of the *VIL2* V1 mRNA was initiated by transcriptional activation.

### The *VIL2* V1 promoter responds to TPA stimulation.

**Fig 3:**
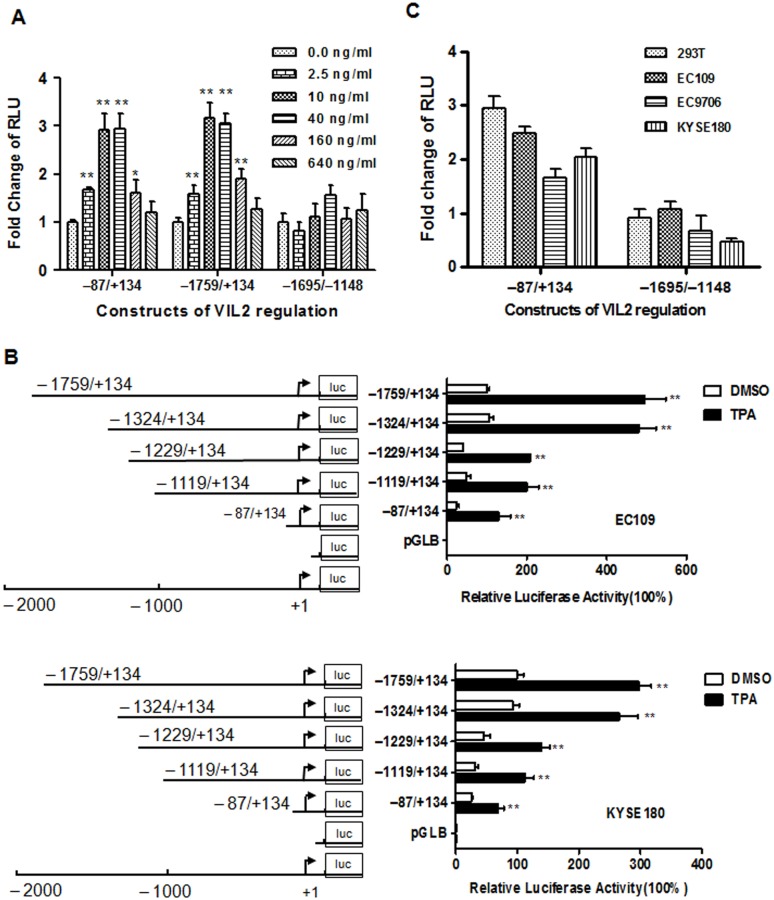
Identification of TPA responsive region. (A) TPA-induced activity of *VIL2* promoter region. Constructs of pGLB-hE(-87/+134) containing *VIL2* V1 promoter region, pGLB-hE(-1759/+134) containing both *VIL2* V1 and V 2 promoter region, pGLB-hE(-1695/-1148) containing *VIL2* V2 promoter region or empty vector pGL3-basic (pGLB) were co-transfected with pRL-TK into EC109 cells simulated with TPA for 24 h before assay the luciferase activity. The *firefly* luciferase activity was normalized to *Renilla* luciferase activity and the relative value from the DMSO-treated control group was considered equal to one arbitrary unit. Each value represents the mean ± SD, n≥3. (B) The schematic of the *VIL2* promoter 5′-deletion constructs used for transient transfections (*Left*) and the relative luciferase activity of whole cell extracts from EC109 and KYSE180 cells transiently transfected with the constructs listed in the left for 24 h and then treated with DMSO or TPA for another 24 h (*Right*). The *firefly* luciferase activity was normalized to *Renilla* luciferase activity and the relative value from the DMSO-treated cells transfected with pGLB-hE (-1759/+134) was set to 100%. Each value represents the mean ± SD, n≥3. (C) TPA-induced activity of *VIL2* promoter region in different cells. pGLB-hE(-87/+134) and pGLB-hE(-1695/-1148) Constructs were used and the relative value from the DMSO-induced control group was considered equal to one arbitrary unit. Each value represents the mean ± SD, n≥3. The data are representative of at least two independent experiments. *p< 0.05 or **p< 0.01.

To identify the region that is responsive to TPA, three reporter constructs containing different fragments of the *VIL2* promoter region (i.e., the full-length cloned fragment (-1759/+134), V1 promoter fragment (-87/+134), or V2 promoter fragment (-1695/-1148)) were transiently transfected into EC109 cells. After the transfected cells were treated with different dosage of TPA for 24 h, luciferase activity was analyzed. The dose-response curve showed increased luciferase activity of the -87/+134 and -1759/+134 constructs starting at 2.5 ng/ml of TPA and reaching a maximum by 10 ng/ml TPA, but not -1695/-1148 construct (Fig 3A). Because the effect of TPA reached a plateau at 10–40 ng/ml, so 10 ng/ml of TPA was used for subsequent experiments. To further confirm the location of the TRE, we transfected a series of 5'-deleted reporter constructs into ESCC cells, and luciferase activity was analyzed after TPA treatment. The -1759/+134 construct containing the full-length *VIL2* promoter region showed the highest luciferase activity, with decreasing activity associated with shorter promoter regions (Fig 3B). Notably, the -87/+134 sequence was still responsive to TPA treatment, suggesting that this fragment contains the TRE(s). These findings were confirmed in other four ESCC cell lines (Fig 3C).Taken together, our results suggest that TREs embedded in the *VIL2* V1 promoter region modulate the V1expression in response to TPA stimulation.

### The Sp1/AP-1 complex is necessary for the response of *VIL2* V1 to TPA

**Fig 4:**
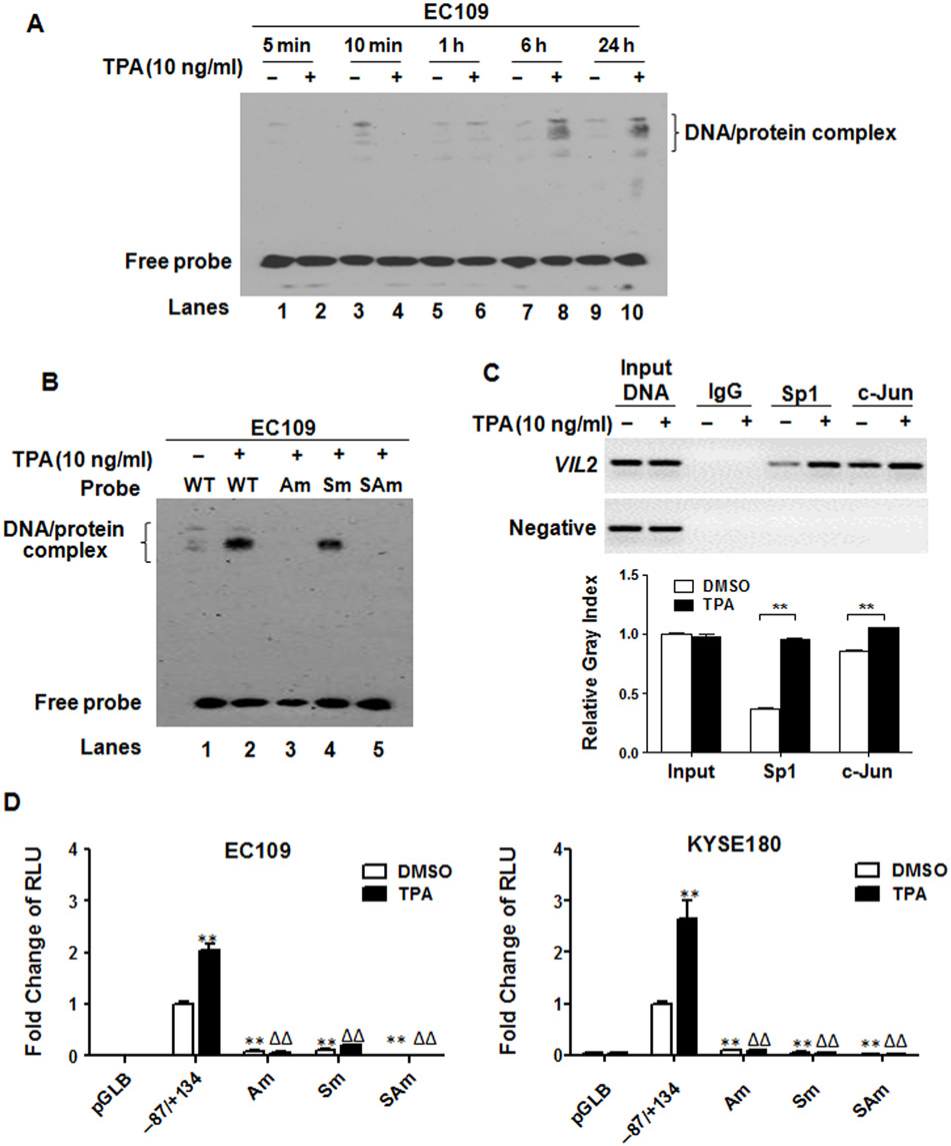
Identification of AP-1/Sp1 composite was TPA response element. (A) EMSA assay of the nuclear extract prepared from EC109 cells bound to the sequence of -87/-46 fragment within the V1 promoter region of *VIL2* after stimulation with DMSO or TPA for indicated time. Probe WT (-87/-46) was labeled with biotin. (B) The specific DNA-protein complex was confirmed by using biotin-labeled Sm, Am, or SAm oligonucleotides covering the same segment with probe WT but containing a substituted sequence (lanes 3 through 5), which are listed in Table 2. The probes were incubated with nuclear extract prepared from EC109 cells treated with DMSO or with TPA for 24 h. (C) Sonicated chromatin isolated from EC109 cells was incubated with antibodies against IgG, Sp1 and c-Jun, respectively. ChIP assay showed that TPA treatment enhanced Sp1 and c-Jun bound to the *VIL2* promoter region. (D) The effect of site-directed mutagenesis of the AP-1/Sp1 binding sites on the *VIL2* V1 promoter activity. The mutagenesis constructs were co-transfected with pRL-TK into EC109 or KYSE180 cells. After transfection for 24 h, the cells were treated with DMSO (control; open bars) or TPA (10 ng/ml; filled bars) for another 24 h before analysis. The *firefly* luciferase activities of mutant constructs were normalized to *Renilla* luciferase activity and shown as a ratio compared to that of the pGLB-hE (-87/+134) construct treated with DMSO, which was set as 1.Each value represents the mean ± SD, n≥3. The data are representative of at least two independent experiments. *p < 0.05 or **p < 0.01, compared to the pGLB-hE (-87/+134) construct treated with DMSO; ^Δ^ *p* < 0.05 or ^ΔΔ^ *p* < 0.01, compared to the pGLB-hE (-87/+134) construct treated with TPA.

It is reported that Sp1 and AP-1 are involved in the alteration of target genes transcription in a TPA-dependent stimulation [25,26]. Our previous work also showed that Sp1/AP-1 binding sites located within the V1 promoter region could regulate the basal transcription of *VIL2* [22]. Therefore, to detect whether the Sp1/AP-1 binding sites located in the *VIL2* V1 promoter region were involved in the TPA-induction, nuclear extracts from EC109 cells treated with TPA were incubated with biotin-labeled oligonucleotides spanning from -87 to -46 bp of the V1 promoter (i.e., WT probe), which contained Sp1 (-75 to -69) and AP-1(-64 to -58) binding site sequences. We found that TPA treatment robustly increased DNA/protein complex formation (Fig 4A). To further investigate whether Sp1 and AP-1 transcription factors were involved in the TPA-induced up-regulation of *VIL2* V1, nuclear extracts were prepared from EC109 cells after treatment with TPA for 24 h and incubated with four biotin-labeled oligonucleotide probes: (1) WT probe containing binding sites of both Sp1 and AP-1, (2) Sm probe containing a mutant Sp1 binding site, (3) Am probe containing a mutant AP-1 binding site, or (4) SAm probe containing double-mutated Sp1 and AP-1 binding sites. Although some DNA/protein complex formed in nuclear extracts incubated with WT probe (Fig 4B, lane 1), TPA treatment markedly increased complex formation (Fig 4B, lane 2). Mutation of the AP-1 binding site (Fig 4B, lane 3) or double mutation of both AP-1 and Sp1 binding sites (Fig 4B, lane 5) abolished DNA/protein complex formation, whereas mutation of the Sp1 binding site only partially reduced complex formation (Fig 4B, lane 4). To validate the specific interaction of Sp1 and AP-1 with *VIL2* promoter, cross-linked chromatin was immunoprecipitated with IgG, anti-Sp1 or anti-c-Jun by ChIP assay, respectively. As shown in Fig 4C, TPA stimulation enhanced the binding of Sp1 and c-Jun to the *VIL2* V1 promoter region. These results suggest that Sp1/AP-1 complex is important for the response of *VIL2* V1 to TPA.

To further confirm the cooperation of Sp1 and AP-1 transcription factors, we analyzed the TPA responsiveness of four reporter constructs: (1) pGLB-hE(-87/+134) containing the V1 promoter, (2) pGLB-hE(-87/+134)Am containing a mutant AP-1 binding site, (3) pGLB-hE(-87/+134)Sm containing a mutant Sp1 binding site, and (4) pGLB-hE(-87/+134)SAm containing both mutant Sp1 and AP-1 binding sites. These constructs were transiently transfected in TPA- or DMSO-treated ESCC cells. Compared with DMSO-treated control group, luciferase activity of the pGLB-hE(-87/+134) construct was significantly increased after TPA treatment (Fig 4D). When either the AP-1 or Sp1 binding site was mutated, however, luciferase activity was reduced by ~90%. When both sites were simultaneously mutated, luciferase activity was almost completely blocked under both DMSO and TPA treatment conditions. Thus, AP-1 and Sp1 binding sites may not only play a critical role in *VIL2* basal promoter activity but also function as TREs within the *VIL2* V1 promoter in ESCC cells.

### TPA promoted cell motility by inducing up-regulation of *VIL2* V1 via the ERK1/2/AP-1/Sp1 signaling

**Fig 5:**
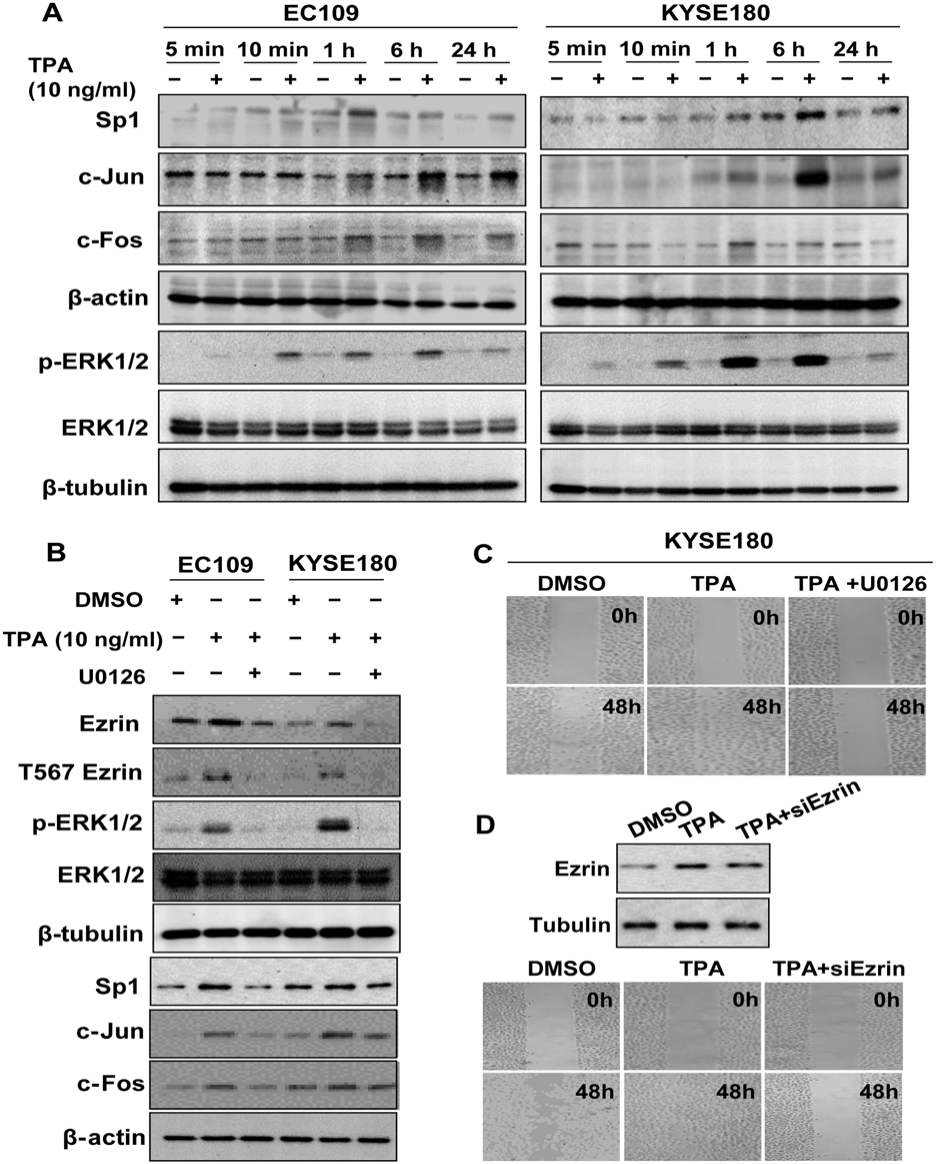
TPA up-regulated *VIL2* V1 transcription via ERK1/2/AP-1/Sp1 signaling. **(A)** The expression of Sp1, c-Jun, c-Fos, phospho-ERK1/2 (Thr202/Tyr204) or total ERK was detected after treated with DMSO or TPA (10 ng/ml) for the indicated time in EC109 and KYSE180 cells. β-actin or β-tubulin was served as a loading control. (B) EC109 and KYSE180 cells were treated with DMSO, TPA (10 ng/ml), or pretreated for 1 h with U0126 (10 μM) before adding TPA, respectively, and alteration of ERK1/2/AP-1/Sp1 signaling was determined. (C) Wound healing assay for the cells treated with TPA (10 ng/ml) or TPA and U0126. (D) Wound healing assay was employed to detect the effect of ezrin knockdown on the TPA-mediated cell migration in KYSE180 cells. Left, western blotting analysis for the ezrin silencing in the TPA-treated cells; Right, cell migration assay.

To explore the involvement of AP-1and Sp1 in TPA-induced expression of *VIL2* V1, EC109 and KYSE180 cells were treated with TPA or DMSO for different durations of time and the whole-cell extracts were analyzed by western blotting. The result showed that Sp1, c-Jun, and c-Fos protein expression levels were up-regulated in a time-dependent manner after TPA treatment (Fig 5A). The expression of Sp1 was peaked at 1 h in EC109 cells but 6 h in KYSE180 cells, c-Jun was peaked at 6h in both cells, and c-Fos was observed by 1 h in KYSE180 cells and 6 h in EC109 cells. To determine whether ERK1/2 pathway took part in this process, we further evaluated the expression levels of ERK1/2 and phosphorylation of ERK1/2 (p-ERK1/2) in TPA-treated cells, and found that p-ERK1/2 was enhanced after TPA treatment in both ESCC cell lines, while total ERK1/2 did not. These preliminary findings suggest that MEK/ERK1/2 signaling mediates the regulation of *VIL2* V1 by TPA (Fig 5A).

As a further confirmation that ERK1/2 signaling participates in the TPA-mediated transcriptional regulation of *VIL2* V1, the cells were pretreated with the specific MEK1/2 inhibitor U0126 for 1 h before TPA was added and the whole-cell extracts were analyzed by western blotting. It showed that U0126 blocked the rapid phosphorylation of ERK1/2, leading to reduced expression of ezrin, and decreased phosphorylation of ezrin at the T567 site (termed here as T567 ezrin), c-Jun, and Sp1 in both cells, but not c-Fos in KYSE180 cells (Fig 5B). Moreover, pretreatment with U0126 decreased the TPA-induced expression of *VIL2* V1 as evidenced by both mRNA expression and luciferase activity (S1 Fig). These results indicate that TPA induces the transcription of *VIL2* V1 in ESCC cells through activating the ERK1/2/AP-1/Sp1 signaling pathway.

Finally, to explore the functional role of TPA-induced ezrin in ESCC cells, we detected the motility of KYSE180 cells after TPA treatment by wound healing assay. Results showed that TPA treatment significantly promoted the motility of ESCC cells, and MEK/ERK1/2 inhibitor U0126 completely reversed this change (Fig 5C). The same results were obtained by using Boyden chamber-based cell migration assay in EC109 cells (S2A Fig). Further analysis revealed that silencing of ezrin by siRNA could also lead to restoration of the TPA-promoted cell migration (Fig 5D; S2B Fig). These data suggested that MEK/ERK1/2/Ezrin might be important effector of TPA-mediated cellular function.

## Discussion

*VIL2* has been found to be not only a key component in tumor metastasis but also a diagnostic marker and therapeutic target for numerous cancers [10,12,14,15,27]. In ESCC, we previously found that overexpression of ezrin promoted cell proliferation and invasiveness [17, 18]. Moreover, Sp1 and AP-1 activated the transcription of human *VIL2* in ESCC cells through binding to the Sp1 site and adjacent AP-1 site of the *VIL2* promoter, respectively, involving the MEK/ERK1/2 signaling pathway [22]. Extracellular factors that induce transcription of *VIL2* in ESCC cells, however, have not been clearly identified. Here, we found that TPA promoted the transcription of human *VIL2* V1 but not V2 in a time-dependent manner in ESCC cells. Furthermore, we found that Sp1 and AP-1 binding sites located in the core promoter region of *VIL2* V1 not only functioned as basal transcriptional elements but also as composite TREs, and the MEK/ERK1/2 signaling pathway enhances the binding of Sp1 and c-Jun to the TRE.

The regulation of gene transcription by combinations of different transcription factors has been widely demonstrated. In particular, Sp1 interacts with different transcription factors such as CCAAT-enhancer-binding proteins (C/EBP) [28], signal transducers and activators of transcription (STAT1) [29], and c-Jun [30], producing synergistic effects on the expression of target genes. Sp1 also cooperates with AP-1 to play a critical role in osteopontin promoter activation downstream of hepatitis C virus-mediated Ca^2+^ signaling [31]. Cooperation between Sp1 and AP-1 has also been found to control loricrin expression [32] and to activate the urokinase-type plasminogen activator gene [33]. Moreover, multiple reports indicate that cooperation between functional Sp1 and AP-1 binding sites does not appear to depend on their spacing [30, 34]. Based on these findings, we suspect that Sp1 may function as a scaffolding protein for recruiting AP-1 components after TPA stimulation to assure the efficient and orderly completion of *VIL2* V1 transcription. The finding that Sp1 directly binds to guanine/cytosine (GC)-rich regions within damaged neuronal endopeptidase promoters, *thus providing* activating transcription factor 3 *(ATF3)/c-Jun/STAT3* with a platform to engage their functional synergy, is also consistent with our hypothesis, as the promoter region of human *VIL2* V1 is GC-rich [30]. AP-1 is a heterodimeric protein containing proteins belonging to the Jun (c-Jun, JunD, and JunB) and Fos (c-Fos, FosB, Fra-1, and Fra-2) families [35,36]. In this study, except for c-Jun and c-Fos, we did not examine other AP-1 components after TPA treatment. A previous study demonstrated that the Jun/Fos heterodimer was more stable than the Jun/Jun homodimer due to its longer half-life and stronger DNA binding affinity [37].

Sp1 and AP-1 are recruited and bind to their respective binding sites within target promoter regions through signal-transducing kinase cascades, leading to the transcription of corresponding genes. Velpula et al. [38] and Zhu et al. [39] found that Sp1 was activated by MEK/ERK for different genes in various cell types. Tissue factor-factor/VIIa/protease-activated receptor 2-induced PKCα and ERK1/2 signaling could phosphorylate c-Jun, which subsequently enhanced c-Jun expression [40]. In human articular cartilage, c-Fos was activated by ERK1/2 signaling upon transactivation of the *IL-11* gene [41]. Also, previous studies showed that activated ERK1/2 phosphorylated Sp1 and AP-1 and enhanced their binding to the *VIL2* promoter, resulting in activation of *VIL2* basal transcription [22]. In the present study, TPA stimulation was also found to activate the MEK/ERK1/2 pathway, leading to the up-regulation of Sp1 and AP-1expression. This suggests that a potential mechanism of TPA-induced transcription of *VIL2* V1 is the activation of the MEK/ERK1/2 pathway, which leads to enhanced expression of Sp1, c-Fos, and c-Jun and promotion of their binding to TRE within the promoter region of *VIL2* V1, ultimately resulting in the up-regulation of *VIL2* V1 in ESCC cells. However, why only *VIL2* V1 but not V2 is responsive to TPA treatment is unclear.

The key point of inducing morbidity and mortality of tumors is their fast migration. TPA has been reported to promote cancer cell motility through regulating the expression and function of S100A14, cyclooxygenase-2 (COX-2) or matrix metalloproteinase-9 (MMP-9) [42,43]. Here, we showed that ERK1/2/AP-1/Sp1/ezrin signaling was involved in TPA-mediated motility of ESCC cells, suggesting that *VIL2* might be an important downstream effector of TPA. Another point should be highlighted was that our results showed TPA not only up-regulated the expression of ezrin, but also increased the phosphorylation of ezrin at T567 site. Phosphorylation of ezrin at T567 was found to be necessary for the activation of ezrin, and was important for the migration process [44, 45]. TPA could induce the activation of certain kinases such as PKCα, which might influence the process of protein phosphorylation [46, 47]. Therefore, we proposed that phosphorylation of ezrin might participate in the TPA-mediated alterations of cell behaviors. Studies for the precise mechanisms are underway.

In summary, we show for the first time that TPA stimulates transcriptional expression of human *VIL2* V1 but not V2 via the ERK1/2-dependent AP-1/Sp1 pathway in ESCC cells, leading to the enhancement of cell motility. Considering the importance of ezrin in the invasion and metastasis of ESCC cells, a full understanding of the mechanisms involved in TPA regulation of ezrin is urgently needed for future clinical applications.

## Supporting Information

S1 Fig MEK/ERK1/2 inhibitor U0126 blocked the TPA-induced increasing of *VIL2* V1. (A) qRT-PCR assay for the message RNA level of *VIL2* V1. The cells were stimulated with TPA as the prior experiment. The relative expression was normalized to the DMSO-treated control. Each value represents the mean ± SD, n≥3. (B) Reporter gene assay of *VIL2* V1 promoter activity. The pGLB-hE(-87/-134) construct was co-transfected with pRL-TK into ESCC cells for 24 h, then the cells were stimulated for 24 h by DMSO, TPA(10 ng/ml), or pretreated for 1 h with U0126 (10 μM) before added TPA. The reporter gene activity was measured. The firefly luciferase activity was normalized to Renilla luciferase activity. Each value represents the mean ± SD, n≥3. The data are representative of at least two independent experiments. *p< 0.05 or **p< 0.01.
(TIF)

S2 Fig TPA increased cell mobility through up-regulation of ezrin via MEK/ERK1/2 pathway in EC109 cells. (A) 24-well Boyden chamber-based cell migration assay was used to determine the alterations of cell migration after being treated with TPA (10 ng/ml) or TPA and U0126. (B) 24-well Boyden chamber-based cell migration assay was employed to detect the effect of ezrin knockdown on the TPA-mediated cell migration. Left, western blotting analysis for the ezrin silencing in the TPA-treated cells; Right, cell migration assay. Representative tumor cells migrated were photographed (40×), data represent mean ± SD of triplicates.
(TIF)

S1 Table Cell lines used in this study and cell culture general information. (PDF)
