# Supplementary figures and images for: 12-O-Tetradecanoylphorbol-13-Acetate Induces Up-Regulated Transcription of Variant 1 but Not Variant 2 of VIL2 in Esophageal Squamous Cell Carcinoma Cells via ERK1/2/AP-1/Sp1 Signaling
Source: PLoS One. 2015 Apr 27;10(4):e0124680. doi: 10.1371/journal.pone.0124680 (PMC4411055; doi:10.1371/journal.pone.0124680)

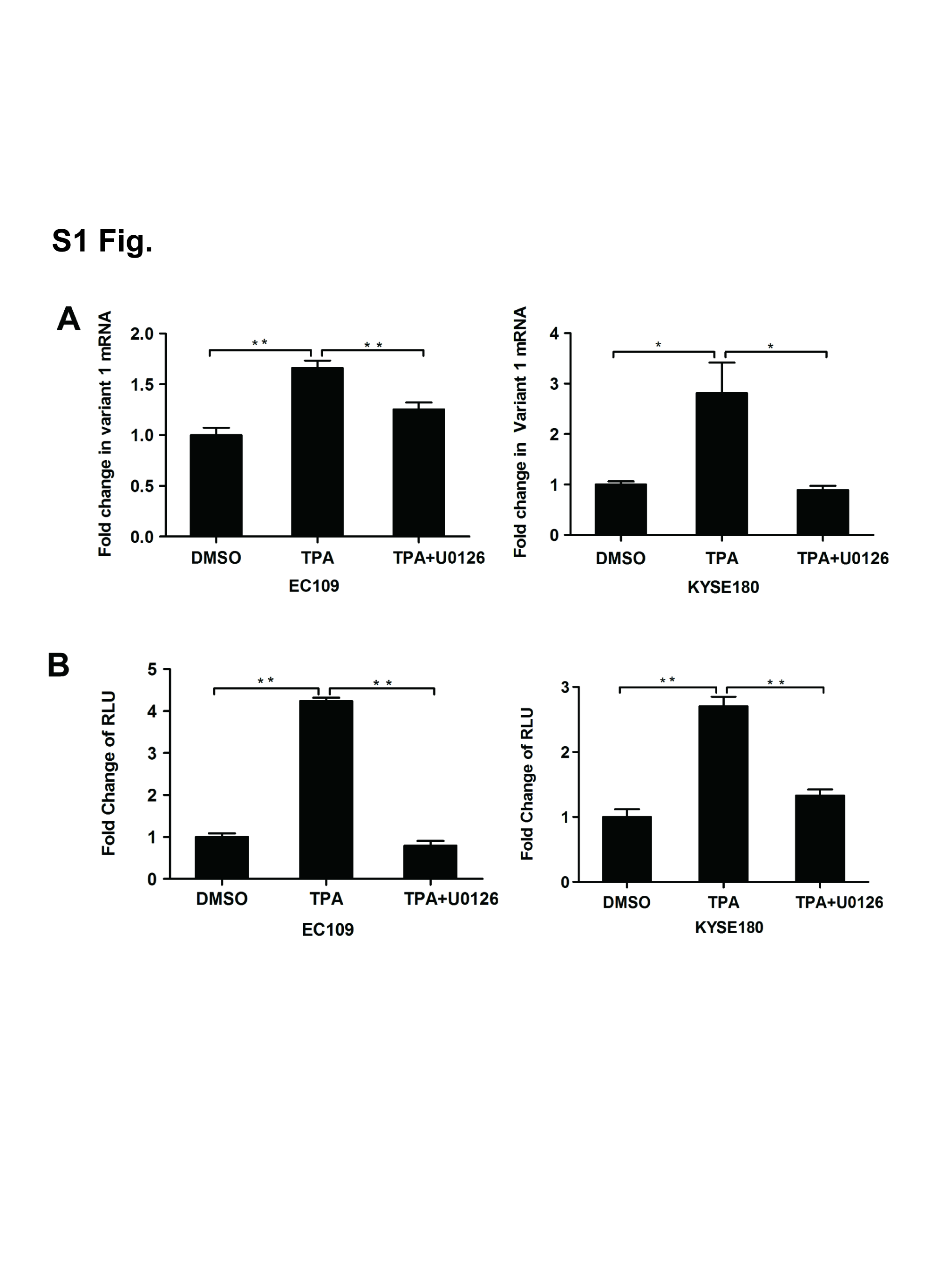

Supplement: S1 Fig — (A) qRT-PCR assay for the message RNA level of VIL2 V1. The cells were stimulated with TPA as the prior experiment. The relative expression was normalized to the DMSO-treated control. Each value represents the mean ± SD, n≥3. (B) Reporter gene assay of VIL2 V1 promoter activity. The pGLB-hE(-87/-134) construct was co-transfected with pRL-TK into ESCC cells for 24 h, then the cells were stimulated for 24 h by DMSO, TPA(10 ng/ml), or pretreated for 1 h with U0126 (10 μM) before added TPA. The reporter gene activity was measured. The firefly luciferase activity was normalized to Renilla luciferase activity. Each value represents the mean ± SD, n≥3. The data are representative of at least two independent experiments. *p< 0.05 or **p< 0.01. (TIF) [file pone.0124680.s001.tif]

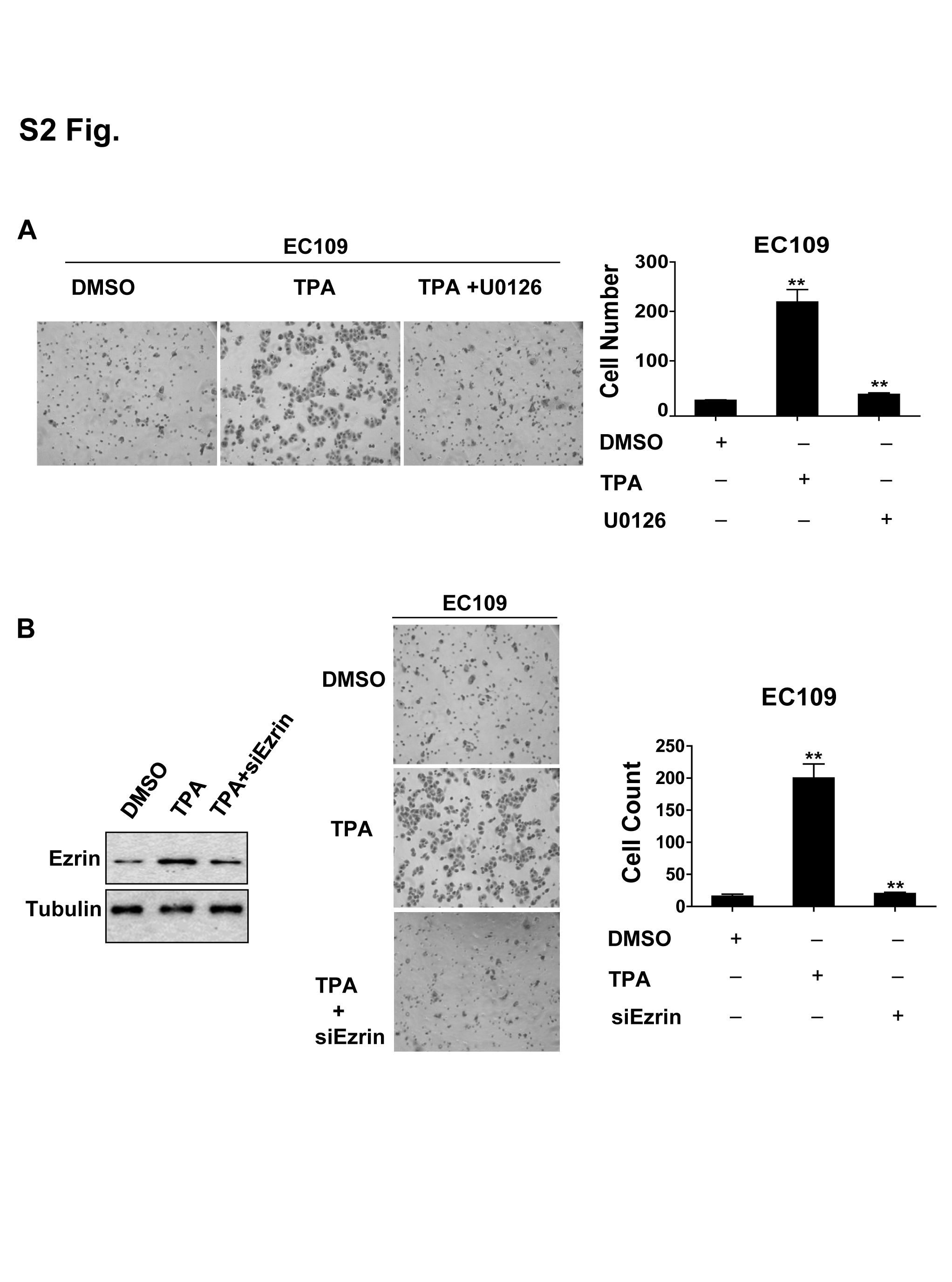

Supplement: S2 Fig — (A) 24-well Boyden chamber-based cell migration assay was used to determine the alterations of cell migration after being treated with TPA (10 ng/ml) or TPA and U0126. (B) 24-well Boyden chamber-based cell migration assay was employed to detect the effect of ezrin knockdown on the TPA-mediated cell migration. Left, western blotting analysis for the ezrin silencing in the TPA-treated cells; Right, cell migration assay. Representative tumor cells migrated were photographed (40×), data represent mean ± SD of triplicates. (TIF) [file pone.0124680.s002.tif]
