# Supplementary material for: 12-O-Tetradecanoylphorbol-13-Acetate Induces Up-Regulated Transcription of Variant 1 but Not Variant 2 of VIL2 in Esophageal Squamous Cell Carcinoma Cells via ERK1/2/AP-1/Sp1 Signaling
Source: PLoS One. 2015 Apr 27;10(4):e0124680. doi: 10.1371/journal.pone.0124680 (PMC4411055; doi:10.1371/journal.pone.0124680)
Supplement: S1 Table — (PDF) [file pone.0124680.s003.pdf]

Table S1. Summary of cell lines and cell culture general information in this study.

Cell line Name	Description	*Culture medium	Cell source	Ref.
SHEE	Immortalized normal esophageal epithelial cells	DMEM /F12 with 10% NBS	established by our laboratory	1
NE2	Immortalized normal esophageal epithelial cells	EpiLife with dKSFM	gift from Professor Sai-Wah Tsao	2
NE3	Immortalized normal esophageal epithelial cells	EpiLife with dKSFM	gift from Professor Sai-Wah Tsao	2
NECA6	Immortalized normal esophageal epithelial cells	EpiLife with dKSFM	gift from Professor Sai-Wah Tsao	2
293T	Human embryonic kidney cells	DMEM with 10% FBS	purchased from Type Culture Collection of Chinese Academy of Sciences	
ChangLiver	Human normal liver cells	DMEM with 10% FBS	purchased from Type Culture Collection of Chinese Academy of Sciences	
EC109	Human esophageal squamous cell carcinoma cells	DMEM with 10% NBS	purchased from Type Culture Collection of Chinese Academy of Sciences	3
EC171	Human esophageal squamous cell carcinoma cells	DMEM with 10% NBS	purchased from Type Culture Collection of Chinese Academy of Sciences	3
EC18	Human esophageal squamous cell carcinoma cells	DMEM with 10% NBS	purchased from Type Culture Collection of Chinese Academy of Sciences	3
EC8712	Human esophageal squamous cell carcinoma cells	DMEM with 10% NBS	purchased from Type Culture Collection of Chinese Academy of Sciences	3
EC9706	Human esophageal squamous cell carcinoma cells	DMEM with 10% NBS	purchased from Type Culture Collection of Chinese Academy of Sciences	3
KYSE70	Human esophageal squamous cell carcinoma cells	1640 with 10% FBS	gift from Professor Ming-Zhou Guo	4
KYSE140	Human esophageal squamous cell carcinoma cells	1640 with 10% FBS	gift from Professor Ming-Zhou Guo	4
KYSE150	Human esophageal squamous cell carcinoma cells	1640 with 10% FBS	gift from Professor Ming-Zhou Guo	4
KYSE180	Human esophageal squamous cell carcinoma cells	1640 with 10% FBS	gift from Professor Ming-Zhou Guo	4
KYSE450	Human esophageal squamous cell carcinoma cells	1640 with 10% FBS	gift from Professor Ming-Zhou Guo	4
KYSE510	Human esophageal squamous cell carcinoma cells	1640 with 10% FBS	gift from Professor Ming-Zhou Guo	4
colo680N	Human esophageal squamous cell carcinoma cells	1640 with 10% FBS	gift from Professor Ming-Zhou Guo	4
TE1	Human esophageal squamous cell carcinoma cells	1640 with 10% FBS	gift from Professor Ming-Zhou Guo	4
TE3	Human esophageal squamous cell carcinoma cells	1640 with 10% FBS	gift from Professor Ming-Zhou Guo	4
SHEEC	Human esophageal squamous cell carcinoma cells	DMEM/ F12 with 10% NBS	established by our laboratory	1,5

SEG1	Human esophageal adenocarcinoma cells	1640 with 10% FBS	gift from Professor Ming-Zhou Guo	
SKGT4				
HepG2	Human liver cancer cells	DMEM with 10% NBCS	purchased from Type Culture Collection of Chinese Academy of Sciences	
SW480	Human colorectal adenocarcinoma cells			
BGC-823	Human gastric adenocarcinoma cells	DMEM with 10% NBCS	gift from Professor You-Yong Lv	6
SGC7901				

[*]Culture medium were supplemented with penicillin-G (100 units/mL) and streptomycin (100 μg/mL); DMEM, Dulbecco's modified Eagle’s medium (Thermo,); NBCS, new-born bovine Serum (Excell biology. Inc. Shanghai, China); [3]EpiLife (Cascade Biologics); dKSFM, defined keratinocyte serum-free medium (GIBCO); FBS, fetal bovine serum (Thermo, Waltham, MA, USA); [6]1640, 1640 medium (Thermo, Waltham, MA, USA); F12, F-12 nutrient mixture (Ham) powder (Thermo,).

References:

1. Shen Z, Cen S, Shen J, et al. Study of immortalization and malignant transformation of human embryonic esophageal epithelial cells induced by HPV18 E6E7. J Cancer Res Clin Oncol 2000;126:589-594.
2. Zhang H, Jin Y, Chen X, et al. Cytogenetic aberrations in immortalization of esophageal epithelial cells. Cancer Genet Cytogenet 2006;165:25-35.
3. Pan QQ. Studies on esophageal cancer cells in vitro. Proc Chin Acad Med Sci Peking Union Med Coll 1989;4:52-57.
4. Shimada Y, Imamura M, Wagata T, et al. Characterization of 21 newly established esophageal cancer cell lines. Cancer 1992;69:277-284.
5. Fang WK, Liao LD, Li LY, et al. Down-regulated desmocollin-2 promotes cell aggressiveness through redistributing adherens junctions and activating beta-catenin signaling in esophageal squamous cell carcinoma. J Pathol. 2013;231:257-270
6. Li N, Guo R, Li W, et al. A proteomic investigation into a human gastric cancer cell line BGC823 treated with diallyl trisulfide. Carcinogenesis 2006;27:1222-1231.
